# Vaccination and Its Results

**Published:** 1867-08

**Authors:** Wm. A. Greene

**Affiliations:** Americus, Ga.


NEW SERIES.

Vol. VIII. AUGUST, 1867. No. 6.

### ORIGINAL COMMUNICATIONS.

### ARTICLE I.

*Vaccination and its Results.* By Dr. Wm. A. Greene, of Americus, Ga.

Since the emancipation of the slaves, and the consequent impossibility of subjecting them to strict quarantine regulations, small pox has prevailed in almost every portion of the South. Communities that had always enjoyed perfect immunity from this loathsome scourge, have had it to prevail epidemically in their midst, and many valuable lives have paid the forfeit of neglect and proper appreciation and enforcement of strict vaccination regulations. In view of this, it certainly behooves every physician to employ active exertions to arouse the people to the great importance of vaccination, and to enlighten their minds as to its harmless and intrinsic value. And to do this, it is necessary the physician should entertain correct and intelligent views as to vaccination and its results, to the end, that much of the prevailing prejudice among the common people (negroes particularly) upon this subject may be dispelled. I am sure there is no subject of so much practical value and importance in medicine, that the people are so poorly informed upon. I propose, in a brief way, to offer a few thoughts upon this all important subject, which, I hope, will attract the attention of those members of the professiou who have not heretofore given it that interest it now deserves.

In the large cities provisions are made for the vaccination of the inhabitants, but in the smaller cities and villages and in the country, there is not only no such provisions made, but the people, and in many instances the physician, manifest a total disregard for, and even *fear* of, applying thisonly sure preventative of the disease. It is not uncommon to hear people say, when urged to be vaccinated, that they "prefer small pox to the risk of vaccination with spurious matter;" and they refer you, perhaps, to our soldiers who suffered so much from vaccination; and, that they knew such an one who had contracted small pox, notwithstanding vaccination, and too, when the vaccination had produced a *tremendous* sore! We sometimes find otherwise intelligent physicians who are at a loss to account for many of the phenomena attending vaccination. This was especially so among the surgons of the army of Northern Virginia, during our occupancy of Fredericksburg, in the winter of 1862–3. Small pox broke out among our troops then, and a general vaccination was wisely ordered. The number of spurious vaccinations were very great, and became alarming, as affecting the efficiency of the army. It was reported by Gen. Lee's Assistant and Inspector General, that in the battle of Chancellorsville we had 5,000 men unfit for duty, because of disability arising from vaccination. Whereupon, Medical Director Guild, a most efficient and watchful officer, issued a circular calling upon Chief Surgeons of Divisions for their opinion of these unpleasant and unusual developments following vaccination,—*unusual*, because of the great numbers. A number and variety of opinions were submitted,—a majority attributing it to spurious matter taken from unhealthy subjects, and the diseases of these subjects propagated through the virus to others. I submitted a report, which, in my opinion, accounted for most of the trouble; a few thoughts of which I will here reproduce, as nearly as I can remember, not having preserved the manuscript of the report, the views therein advanced, applying strictly to private as to military practice and experience. I do not believe that chronic diseases or diseases latent or undeveloped in the system, can be propagated through vaccine virus. They certainly are not likely to be. But they must be in a state of *activity* or *excitement*, or in the *acute* stage. I have often taken the matter from a subject laboring under *chronic* eruptive disease, and vaccinated healthy subjects with good result. I have, also, taken the matter from a ripe pustule of a constitutional syphilitic subject, and produced a good result on a healthy subject, without any sign of the syphilitic poison appearing. Since vaccination is adopted almost universally in every civilized country, if it were possible for disease to be thus transmitted, is it not reasonable to suppose that a large proportion of the people would be more or less diseased who are vaccinated; and, more especially is this to be presumed, since vaccine matter is said not be deteriorated by frequent transmissions; and what a variety and intensity of disease must accumulate in this matter, which, in all probability, had passed through so many systems. Therefore, much of the dread arising from this cause is ill-founded.

In all these cases of spurious vaccination above referred to in the army of Northern Virginia, there were eruptions of various kinds; and they were not confined to the soldiers of this army, but prevailed among civilians in every section of the country, so far as my observation or knowledge extended. The cause of this eruption, I am sure, can never be arrived at from the study of statistical tables, no matter how carefully compiled; nor from any pathological state of the system, as to the character of the virus, with any degree of certainty. There are, however, certain well established facts and legitimate deductions from plainly defined pathological developments, which will enable us to arrive at quite satisfactory results.

1. It is a well established fact, that primary vaccination results in less *local*, but more constitutional excitement than re-vaccination.

2. That re-vaccination produces more soreness in the extremity operated on, and causes much more frequent irritation and enlargement of the Lymphatic glands than primary operations.

From these facts may be drawn the conclusion,—

A. That when the system is fully susceptible of the impressions of the virus, not having been once subjected to its influence, it receives it more kindly, and diffuses it more generally, and, hence, produces less local and consequently less morbid action.

B. When there is any obstruction to the imbibition of the virus, either from previous vaccination, innoculation, or present morbid condition of the system, it is expended in the glands and tissues of the neighboring parts, and produces greater local irritation.

3. Decomposed animal matter, when introduced into the system through a puncture or incision, in very small quantities, will produce much local irritation, and frequently an enlargement of the glands, with copious suppuration, as is evidenced in the absorption of the exudation from "toe itch," or the violent malignant erysipelas which follows cuts and wounds of any kind in dissection.

From which we infer,—

C. That a vaccine scab which has been *bruised*, so as to prevent its healthful development, may contain animal matter in such a stage of decomposition as to result in the production of lichen, bullæ, vesiculæ, erysipelas, etc., when introduced into the system.

Among the troops, crowded as they were in small tents, and exposed to injury from the handling of their guns and accoutrements, in drilling, and on guard and police duty, *bruising* of the *scab* must have been of frequent occurrence, thus preventing its healthful development, and impregnating it with decomposed animal matter. It was an every day occurrence for these men to vaccinate each other from their own arms, and thus was produced much of the unpleasant developments arising from vaccination. This was not confined to the troops, but might, and did occur among civilians in private practice. We thus frequently attribute an unpleasant vaccination to virus taken from an unhealthy subject, when in all probability, it is the result of vitiated matter from a scab that has been *bruised*, and whose healthful development has been thus perverted.

4. Again, we know that in certain "constitutional states of the atmosphere," the system is so influenced, and fluids are so "concocted" as Sydenhand would term it, that the slightest local irritation will result in the development of bullæ, vesiculæ, and sometimes of phlegmonous erysipelas.

From which may be inferred,—

That in certain states of the atmosphere and conditions of the system, vaccination, with the *purest* virus, will certainly produce much local, as often constitutional aberration.

I remember that the winter of 1862 and 1863, and the following spring, when these unpleasant developments from vaccination were most prevalent, was characterized as peculiarly favorable to the development of pustulæ, bullæ, erysipelas, etc. I noticed this fact not only among the troops around Fredericksburg, but among civilians generally throughout the country, so far as my observation extended. Hence we infer that much of the unpleasant development resulting from vaccination, is the result of the state of the atmosphere, producing a morbid condition of the animal tissues and fluids. This conclusion is strengthened by the consideration that these morbid developments are as common in private practice as in the army, as well as the additional fact, that from the *same scab* one has gone through a healthful vaccination, another has suffered from bullæ, another from lichen, and another from erysipelas. This I think accounts for a large number of such cases, while those resulting from spurious or vitiated matter are exceptional, and can be explained by fact 3.

In all such cases I have found the application of liquor acetatis plumbi to the parts, and full purgative with vegetable and saline cathartics, speedily affects a cure. Diet should be spare, cooling, and very simple—milk, rice, gruel, etc.

